# The real-world burden of atopic dermatitis: MEASURE-AD results from Brazil, Mexico, and Argentina^☆^

**DOI:** 10.1016/j.abd.2024.05.011

**Published:** 2025-03-20

**Authors:** Catalina Rincón Pérez, Valeria Aoki, Roberta F. Criado, Martti Antila, Maria Valeria Angles, Tania Ferreira Cestari, Delfina Guadalupe Villanueva Quintero, Gabriel Magariños, Carla Castro, Adriana López Tello-Santillán, Magda Weber, Daniel Lorenzini, Caio Cesar Silva de Castro, Jorge Maspero, Linda García-Hidalgo, Limei Zhou, Shereen Hammad, Lucila de Campos, Tatiane Cristina Rodrigues, Carolina Arzelán, Paula C. Luna

**Affiliations:** a GA^2^LEN Atopic Dermatitis Center of Reference and Excellence, Medical Specialty Unit, Secretaria de la Defensa Nacional, CDMX, Mexico; b Department of Dermatology, Faculty of Medicine, Faculdade de Medicina da Universidade de São Paulo, São Paulo, SP, Brazil; c Department of Dermatology, Faculty of Medicine, Centro Universitário Saúde ABC, Santo André, SP, Brazil; d Clínica de Alergía, Sorocaba, SP, Brazil; e Department of Dermatology, Hospital Italiano de Buenos Aires, Ciudad Autónoma de Buenos Aires, Argentina; f Department of Dermatology, Hospital de Clínicas de Porto Alegre, Universidade Federal do Rio Grande do Sul, Porto Alegre, RS, Brazil; g Grupo Clínico CATEI (Centro de Atención en Enfermedades Inflamatorias) Sociedad Civil, Guadalajara, Jal, Mexico; h Clinical Research, Psoriahue Medicina Interdisciplinaria, Ciudad Autónoma de Buenos Aires, Argentina; i NEKI Servicios Médicos, Vicente Guerreo, Toluca, Méx, Mexico; j Department of Dermatology, Santa Casa de Misericordia de Porto Alegre, Porto Alegre, RS, Brazil; k Escola de Medicina, Pontificia Universidade Católica do Paraná, Curitiba, PR, Brazil; l Allergy and Respiratory Medicine, Fundación CIDEA, Buenos Aires, Argentina; m Médico adscrito al Departamento de Dermatología en el Instituto Nacional de Ciencias Médicas y Nutrición Salvador Zubirán, Ciudad de México, Mexico; n Data and Statistical Sciences, AbbVie Inc., North Chicago, Illinois, United States; o Intercontinental Dermatology, AbbVie Biopharmaceuticals GmbH, Dubai, United Arab Emirates; p Imunology (Dermatology), AbbVie Brasil, São Paulo, Brazil; q Intercontinental Dermatology, AbbVie Brasil, São Paulo, SP, Brazil; r Dermatology, AbbVie S.A., Ciudad Autónoma de Buenos Aires, Argentina; s Instituto de Neumonologia y Dermatologia, Hospital Alemán, Ciudad Autónoma de Buenos Aires, Argentina

**Keywords:** Atopic dermatitis, Cost of illness, Latin America, Therapeutics

## Abstract

**Background:**

Atopic dermatitis (AD) burden increases with disease severity.

**Objective:**

Characterize the real-world burden of AD in Brazil, Mexico, and Argentina.

**Methods:**

MEASURE-AD enrolled patients (≥12-years old) with moderate to severe AD receiving or candidates for systemic therapy between December 2019-December 2020. Patient characteristics, treatments, and outcomes were recorded during one office visit. Primary outcome measures included worst itch/past 24 hours (Worst Pruritus Numerical Rating Scale [WP-NRS]), quality of life (QoL, Dermatology Life Quality Index [DLQI] and Children's DLQI [CDLQI]).

**Results:**

Of 180 patients (adults, n = 157; adolescents, n = 23), 52.2% were male, the mean (SD) age was 33.8 (17.0) years, and all were receiving AD treatment (65.6% systemic therapy). Severe pruritus (WP-NRS ≥ 7) was reported by 54.4% (adults, 57.3%; adolescents, 34.8%). A very/extremely large effect on QoL (DLQI/CDLQI ≥ 11) was reported among 50.0% of patients ≥ 16 years old and 42.9% of patients 12–15 years old. The mean Eczema Area Severity Index (EASI) was 17.0 (adults, 17.7; adolescents, 12.4); 3.9% of patients had clear skin (EASI 0) and 26.7% had severe AD (EASI 23–72). Over the previous 6 months, 0, 1–2, 3–4, 5–6, and > 6 flares were reported by 8.3%, 27.2%, 31.1%, 11.7%, and 15.6% of patients, respectively. On average, flares lasted 15.2 days (adults, 15.9 days; adolescents, 11.1 days).

**Study limitations:**

Patient self-reported information and recall during one office visit.

**Conclusions:**

Despite treatment, disease severity and impact on QoL were high, suggesting that AD is not adequately controlled in all patients, highlighting a considerable unmet need for effective treatments to reduce AD burden.

## Introduction

Atopic dermatitis (AD), also known as atopic eczema, is a chronic, relapsing, inflammatory skin disease that often has a negative physical, psychological, and socioeconomic effect on patients’ lives.^1^, ^2^ The burden of AD increases with disease severity and with the repeated occurrence of disease flares, adversely affecting patient Quality of Life (QoL).^2^, ^3^, ^4^

The management of AD aims to reduce symptoms (e.g., itch, pain, sleep disturbance), and inflammation, induce skin clearance, improve QoL, and control the disease over the long term.^1^ Treatment strategy varies by disease severity, patient age, and country and can be influenced by treatment accessibility and local recommendations as well as differences in healthcare resources across countries and geographic regions.^5^, ^6^, ^7^, ^8^, ^9^

Few studies have reported AD disease severity and treatment patterns in Latin America. The overall prevalence of AD among children and adolescents from Latin America has been reported to range from 2.8% to 24.6% across countries,^10^, ^11^ whereas, generally, a lower prevalence has been reported among adults (Argentina, 3%–5%;^12^, ^13^ Brazil, Mexico, and Colombia, 2%–10%).^13^, ^14^ In three studies conducted in Brazil and Colombia, approximately two-thirds of patients were reported to have moderate to severe disease (65%–87% across studies and assessment methods).^15^, ^16^, ^17^ In the same studies, the most common treatments included topical corticosteroids and oral antihistamines followed by systemic corticosteroids.^15^, ^16^, ^17^ The use of biologic therapies remained low (approximately 10% of patients).^16^, ^17^ A negative impact on QoL, work productivity, and direct and indirect costs has also been reported.^15^, ^16^, ^18^, ^19^ However, gaps remain in the authors’ understanding of the effect of AD on the lives of patients with moderate to severe disease in Latin America.

The objective of this analysis of the MEASURE-AD study was to assess the physical, psychological, and socioeconomic burden of disease, treatment patterns, and Healthcare Resource Utilization (HCRU) in adolescent and adult patients with moderate to severe AD in Latin America who were receiving or were candidates for systemic therapy.

## Methods

### Study design and participants

MEASURE-AD was a cross-sectional, multicounty, observational cohort study conducted in 28 countries across Western Europe/Canada, Asia/Australasia, Eastern Europe/Middle East, and Latin America.^20^ Reported here are results for patients enrolled in MEASURE-AD in three Latin American countries (Brazil, Mexico, and Argentina).

The study design has been reported previously.^20^ Briefly, MEASURE-AD enrolled adults (≥18 years-old) and adolescents (12–17 years-old) with AD during a routine dermatology clinic or office visit between December 2019 and December 2020. Patients who had a physician-confirmed diagnosis of AD, had physician-assessed moderate to severe disease, and were either current candidates for systemic therapy for AD according to the healthcare professional, or were currently receiving systemic therapy for AD were included in the study. Additionally, 6 months of medication history was required. Patients needed to provide a patient authorization form or disclose personal health information and give informed consent (with parental support as required). Notifications/submissions to the responsible ethics committees, health institutions, and/or competent authorities were performed as required by applicable local laws and regulations. Patients were excluded if they were currently participating in an interventional clinical trial (participation in another non-interventional study or registry was not an exclusion criterion).

### Endpoints

The primary endpoints were the worst itch within the past 24 hours assessed using Worst Pruritus Numeric Rating Scale (WP-NRS; score range 0–10) and QoL using Dermatology Life Quality Index (DLQI; assessed in patients aged ≥ 16 years; score range 0–30) or Children’s DLQI (CDLQI; assessed in patients aged 12–15 years; score range 0–30); higher score indicates greater itch or lower QoL.

In addition, the following secondary endpoints were assessed: Patient Oriented Eczema Measurement (POEM; score range 0–28), patient-assessed disease control (using the Inadequately Controlled AD Questionnaire based on the statement, “I feel my current treatments are effective in controlling my atopic dermatitis”, on a 5-point scale ranging from “completely disagree” to “completely agree”), SCORing Atopic Dermatitis (SCORAD; score range 0–103), Validated Investigator Global Assessment for Atopic Dermatitis (vIGA-AD; score range 0–4), body surface area involvement (score range 0%–100%), and Eczema Area and Severity Index (EASI; score range 0–72). Frequency and duration of disease flares within the last 6 months were evaluated based on patient self-report (flare was defined as a sudden worsening of AD with a need for treatment escalation or a need to visit a healthcare provider because of AD worsening). The effect of AD on sleep was also evaluated (during the past week: hours of sleep per night, minutes until falling asleep, and sleep interfering with daily function).

Other patient-reported endpoints included 5D-Pruritus (score range 5–25), Atopic Dermatitis Impact Scale (ADerm-IS), Atopic Dermatitis Symptom Scale (ADerm-SS), Hospital Anxiety and Depression Scale (HADS), including HADS anxiety (HADS-A; score range 0–21) and HADS depression (HADS-D; score range 0–21) subscales, and Short Form-12 Health Survey (SF-12; score range 0–100) for adults and Short Form-10 Health Survey (SF-10; score range 0–100) for adolescents. In addition, Work Productivity and Activity Impairment due to AD (WPAI-AD) and HCRU (number of healthcare visits and the number of acute care visits in the last 6 months due to AD and out-of-pocket expenses for specified healthcare aspects for AD) were assessed.

### Statistical analyses

Data were collected during a single visit. In addition, retrospective data previously collected from healthcare providers were reported. All analyses were based on observed data. Continuous data were descriptively characterized using mean, Standard Deviation (SD) and median, Interquartile Range (IQR). Categorical data were characterized descriptively using frequency distributions (i.e., number and percentage of patients).

Subgroup analyses by EASI disease severity levels (clear, 0; mild, 0.1–5.9; moderate, 6.0–22.9; and severe, 23.0–72.0),^21^ systemic therapy use (yes/no), dupilumab use (yes/other systemic), DLQI effect levels (no effect, 0–1; small, 2–5; moderate, 6–10; very large, 11–20; and extremely large, 21–30), POEM disease severity levels (clear or almost clear, 0–2; mild, 3–7; moderate, 8–16; severe, 17–24; and very severe, 25–28),^22^ and AD Symptom Scale Total Symptom Score–7 (ADerm-SS TSS-7) score category (< 28 vs. ≥ 28) were conducted.

Differences among subgroups were statistically compared; a Kruskal-Wallis test was used for continuous variables and a Chi-Square test for categorical variables. All statistical analyses were carried out by means of the SAS® package version 9.4 (SAS, Cary, NC, USA).

## Results

The MEASURE-AD Latin American population (Brazil, Mexico, Argentina) consisted of 180 patients (adults, n = 157; adolescents, n = 23). Mean (SD) age was 33.8 (17.0) years, and 52.2% of patients were male (Table 1). At the time of the study visit, patients had a history of AD averaging 16.5 years (adults, 17.2 years; adolescents, 11.6 years).

**Table 1:** Baseline patient demographics and characteristics among patients from Brazil, Mexico, and Argentina.

	Total population (n = 180)	Adults (n = 157)	Adolescents (n = 23)
Age, mean (SD)/median (IQR), y	33.8 (17.0)	36.6 (16.4)	14.8 (1.6)
	28.0 (21.0–43.0)	32.0 (23.0–46.0)	15.0 (13.0–16.0)
Male sex, n (%)	94 (52.2)	82 (52.2)	12 (52.2)
BMI, mean (SD)/median (IQR), kg/m^2^	25.9 (4.7)	26.3 (4.5)	22.9 (5.0)
	25.2 (22.9–28.6)	25.4 (23.3–28.8)	21.9 (19.6–25.8)
Duration of AD, mean (SD)/median (IQR), y	16.5 (12.1)	17.2 (12.7)	11.6 (4.1)
	15.1 (6.5–23.7)	16.7 (6.1–24.6)	12.7 (9.4–14.4)
Inadequately controlled AD, n (%)	45 (25.0)	42 (26.8)	3 (13.0)
Time from AD diagnosis to first therapy, mean (SD)/median (IQR), y	9.1 (10.8)	9.3 (11.5)	7.2 (3.6)
	5.1 (0.3–16.1)	5.0 (0.0–16.3)	7.7 (4.0–9.7)
	n = 52	n = 45	n = 7
Time from AD diagnosis to first systemic therapy, mean (SD)/median (IQR), y	10.3 (10.6)	10.8 (11.1)	7.3 (5.1)
	8.4 (1.2–17.0)	8.2 (1.0–18.4)	8.9 (2.4–12.5)
	n = 143	n = 124	n = 19
Continuous systemic therapy over previous 12 mo, n (%)	34 (18.9)	29 (18.5)	5 (21.7)
Current therapy, n (%)	180 (100.0)	157 (100.0)	23 (100.0)
Systemic therapy, alone or in combination	118 (65.6)	101 (64.3)	17 (73.9)
Systemic corticosteroids	41 (34.7)	38 (37.6)	3 (17.6)
Methotrexate	39 (33.1)	32 (31.7)	7 (41.2)
Dupilumab	29 (24.6)	24 (23.8)	5 (29.4)
Cyclosporine	20 (16.9)	19 (18.8)	1 (5.9)
Azathioprine	2 (1.7)	1 (1.0)	1 (5.9)
Systemic therapy alone	22 (12.2)	20 (12.7)	2 (8.7)
Systemic corticosteroids	41 (22.8)	38 (24.2)	3 (13.0)
Methotrexate	39 (21.7)	32 (20.4)	7 (30.4)
Dupilumab	29 (16.1)	24 (15.3)	5 (21.7)
Cyclosporine	20 (11.1)	19 (12.1)	1 (4.3)
Azathioprine	2 (1.1)	1 (0.6)	1 (4.3)
Topical therapy, alone or in combination	134 (74.4)	117 (74.5)	17 (73.9)
Topical therapy, alone	11 (6.1)	10 (6.4)	1 (4.3)
TCS or TCI alone	6 (3.3)	6 (3.8)	0 (0.0)
Previous systemic therapy, n (%)	166 (92.2)	145 (92.4)	21 (91.3)
Systemic therapy, alone or in combination	92 (51.1)	85 (54.1)	7 (30.4)
Systemic therapy alone	21 (11.7)	19 (12.1)	2 (8.7)
Dupilumab, alone or in combination	2 (1.1)	1 (0.6)	1 (4.3)
Dupilumab alone	0	0	0

AD, Atopic Dermatitis; BMI, Body Mass Index; IQR, Interquartile Range; SD, Standard Deviation; TCI, Topical Calcineurin Inhibitor; TCS, Topical Corticosteroid.

All patients were receiving AD treatment, including 74.4% receiving topical therapy alone or in combination (6.1% topical therapy alone). Although all patients were eligible for systemic treatment, only 65.6% were receiving systemic therapy alone or in combination and 12.2% were receiving systemic therapy alone; 18.9% of patients had continuous systemic therapy over the last 12 months (Table 1).

Among the 118 patients receiving systemic therapies, the most common were systemic corticosteroids (34.7%), methotrexate (33.1%), dupilumab (24.6%), and cyclosporine (16.9%); among all 180 patients, the usage was 22.8% for systemic corticosteroids, 21.7% for methotrexate, 16.1% for dupilumab (15.3% among adults), and 11.1% for cyclosporine. The mean (SD) time between AD diagnosis and until first administration of systemic treatment was 10.3 (10.6) years (adults, 10.8 [11.1] years; adolescents, 7.3 [5.1] years). Approximately one-quarter of patients overall (26.8% in the adult population and 13.0% of the adolescent population) reported that they had inadequately controlled disease.

### Primary endpoints: Itch and QoL

**Fig. 1:**
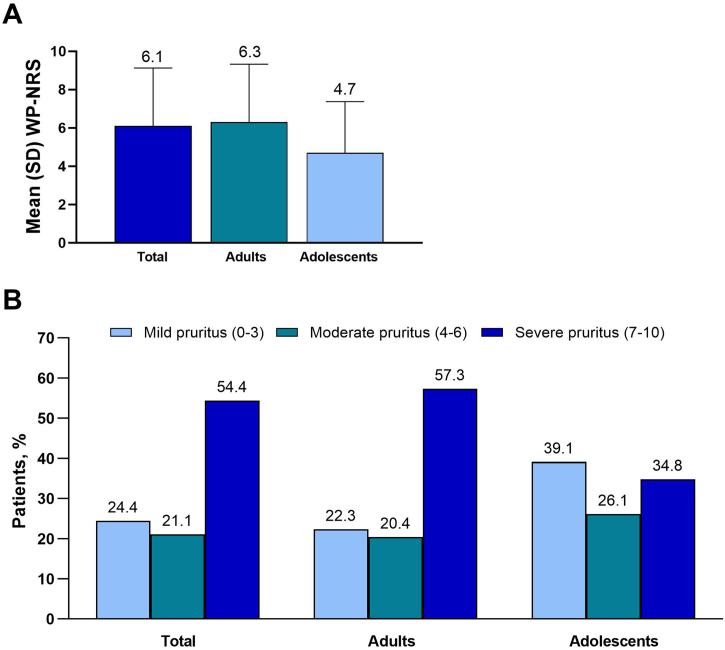
Primary endpoint (A) mean (SD) WP-NRS and (B) proportion of patients in WP-NRS categories. WP-NRS, Worst Pruritus Numeric Rating Scale.

**Fig. 2:**
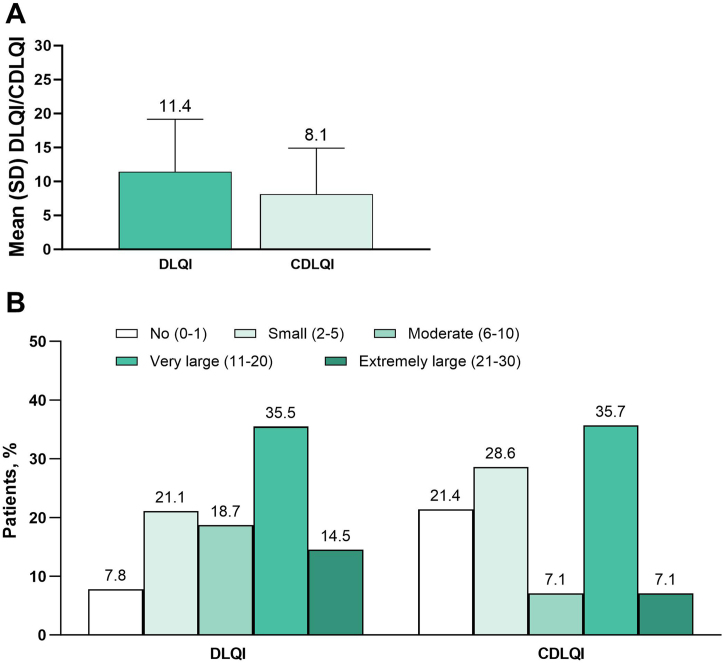
Primary endpoint (A) mean (SD) DLQI/CDLQI and (B) proportion of patients in DLQI/CDLQI categories. CDLQI, Children’s Dermatology Life Quality Index; DLQI, Dermatology Life Quality Index.

The mean WP-NRS score was 6.1, and median score was 7 (adults, 6.3 and 7, respectively; adolescents, 4.7 and 5) (Fig. 1A). Severe pruritus (WP-NRS ≥7) was reported by 54.4% of patients (adults, 57.3%; adolescents, 34.8%) (Fig. 1B). The mean DLQI was 11.4, and the mean CDLQI was 8.1 (Fig. 2A). A very or extremely large effect on QoL (DLQI or CDLQI ≥ 11) was reported by 50.0% of patients ≥ 16 years old and 42.9% of patients 12 to 15 years old (Fig. 2B).

### Secondary endpoints

Secondary endpoints showed a similar burden of disease for both the adult and adolescent populations (Table 2). The mean POEM was 15.7 (adults, 16.3; adolescents, 11.4). The total population reported a mean of 6.1 hours slept per night. Overall, 48.3% of patients reported sleep problems that interfered with daily function over the past week as occurring “somewhat to very much”. The mean SCORAD score was 52.5 and was higher among adults (54.3) compared with adolescents (40.8). A total of 9.4% of patients had mild AD (SCORAD < 25), 31.7% moderate AD (SCORAD 25–50), and 58.3% severe AD (SCORAD > 50).

**Table 2:** Clinical Burden of AD.

	Total population (n = 180)	Adults (n = 157)	Adolescents (n = 23)
POEM, mean (SD)/median (IQR)	15.7 (7.4)	16.3 (7.3)	11.4 (7.0)
	17.0 (10.0–21.0)	17.0 (11.0–22.0)	11.0 (6.0–11.0)
Average hours of sleep per night in the past week, mean (SD)/median (IQR)	6.1 (1.9)	5.9 (1.9)	7.4 (1.4)
	6.0 (5.0–8.0)	6.0 (5.0–7.0)	8.0 (6.0–8.0)
Average minutes needed to fall asleep per night in the past week, mean (SD)/median (IQR)	43.2 (50.3)	43.5 (49.1)	41.4 (58.8)
	30.0 (17.5–60.0)	30.0 (20.0–60.0)	20.0 (10.0–60.0)
Sleep problems interfered with daily function over the past week, n (%)			
I do not have sleep problems	14 (7.8)	11 (7.0)	3 (13.0)
Not at all	48 (26.7)	37 (23.6)	11 (47.8)
A little	31 (17.2)	26 (16.6)	5 (21.7)
Somewhat	28 (15.6)	28 (17.8)	0 (0.0)
Much	31 (17.2)	30 (19.1)	1 (4.3)
Very much	28 (15.6)	25 (15.9)	3 (13.0)
SCORAD, mean (SD)/median (IQR)	52.5 (20.7)	54.3 (19.9)	40.8 (22.5)
	55.3 (40.5–67.1)	55.9 (43.2–68.8)	44.0 (30.8–60.9)
	n = 179	n = 156	
SCORAD categories, n (%)			
Mild (<25.0)	17 (9.4)	12 (7.6)	5 (21.7)
Moderate (25.0–50.0)	57 (31.7)	49 (31.2)	8 (34.8)
Severe (>50.0)	105 (58.3)	95 (60.5)	10 (43.5)
vIGA-AD, mean (SD)/median (IQR)	2.9 (1.0)	3.0 (0.9)	2.5 (1.3)
	3.0 (3.0–4.0)	3.0 (3.0–4.0)	3.0 (1.0–3.0)
vIGA-AD categories, n (%)			
Clear (0)	6 (3.3)	4 (2.5)	2 (8.7)
Almost clear (1)	12 (6.7)	8 (5.1)	4 (17.4)
Mild (2)	17 (9.4)	15 (9.6)	2 (8.7)
Moderate (3)	98 (54.4)	88 (56.1)	10 (43.5)
Severe (4)	47 (26.1)	42 (26.8)	5 (21.7)
Body surface area, mean (SD)/median (IQR), %	26.5 (21.3)	27.2 (21.5)	21.4 (19.0)
	21.5 (10.0–40.0)	22.0 (10.0–40.0)	20.0 (6.0–35.0)
EASI, mean (SD)/median (IQR)	17.0 (11.5)	17.7 (11.4)	12.4 (10.7)
	15.0 (8.9–23.6)	15.9 (9.2–24.6)	9.8 (4.4–18.0)
EASI categories, n (%)			
Clear (0)	7 (3.9)	4 (2.5)	3 (13.0)
Mild (0.1–5.9)	18 (10.0)	14 (8.9)	4 (17.4)
Moderate (6.0–22.9)	107 (59.4)	95 (60.5)	12 (52.2)
Severe (23.0–72.0)	48 (26.7)	44 (28.0)	4 (17.4)
Number of flares in the past 6-months, mean (range)/median (IQR)	5.0 (0–50)	4.9 (0–50)	5.8 (0–30)
	3.0 (2.0–6.0)	3.0 (2.0–6.0)	3.0 (2.0–4.0)
	n = 169	n = 148	n = 21
Number of flares in past 6-months, n (%)			
0	15 (8.3)	12 (7.6)	3 (13.0)
1–2	49 (27.2)	44 (28.0)	5 (21.7)
3–4	56 (31.1)	48 (30.6)	8 (34.8)
5–6	21 (11.7)	20 (12.7)	1 (4.3)
> 6	28 (15.6)	24 (15.3)	4 (17.4)
Missing	11 (6.1)	9 (5.7)	2 (8.7)
Average duration of flares in past 6-months, mean (range)/median (IQR), d	15.2 (0–180)	15.9 (0–180)	11.1 (0–90)
	7.0 (4.0–15.0)	10.0 (5.0–15.0)	7.0 (3.0–8.0)
	n = 172	n = 149	
Average duration of flares in past 6-months, n (%), d			
≤ 2	28 (15.6)	23 (14.6)	5 (21.7)
3–7	60 (33.3)	48 (30.6)	12 (52.2)
8–14	26 (14.4)	24 (15.3)	2 (8.7)
≥ 15	58 (32.2)	54 (34.4)	4 (17.4)
Missing	8 (4.4)	8 (5.1)	0
5D-Pruritus score, mean (SD)/median (IQR)	16.2 (4.5)	16.5 (4.3)	13.9 (4.6)
	17.0 (13.0–20.0)	17.0 (13.0–20.0)	12.5 (11.0–15.0)
	n = 176	n = 154	n = 22
Total ADerm-IS, mean (SD)/median (IQR)	42.3 (30.7)	45.0 (30.4)	23.5 (26.7)
	40.0 (13.0–69.0)	43.0 (18.0–71.0)	11.0 (6.0–34.0)
	n = 177	n = 155	n = 22
ADerm-IS Sleep Domain, mean (SD)/median (IQR)	13.5 (10.6)	14.3 (10.6)	8.0 (9.3)
	13.0 (2.0–24.0)	15.0 (4.0–24.0)	3.0 (0.0–15.0)
	n = 179		n = 22
ADerm-SS TSS-7, mean (SD)/median (IQR)	34.2 (21.1)	36.2 (20.7)	20.3 (18.9)
	31.0 (17.5–53.0)	35.0 (20.0–54.0)	14.0 (6.0–26.0)
ADerm-SS TSS-11, mean (SD)/median (IQR)	49.2 (31.6)	51.8 (31.1)	31.3 (29.3)
	44.0 (24.0–76.0)	49.0 (29.0–77.0)	26.0 (9.0–44.0)
	n = 177	n = 154	
ADerm-SS Skin pain, mean (SD)/median (IQR)	4.4 (3.7)	4.6 (3.8)	2.9 (3.1)
	4.0 (1.0–8.0)	5.0 (1.0–8.0)	2.0 (0.0–5.0)
SF-12 PCS, mean (SD)/median (IQR)	NA	48.6 (9.0)	NA
		50.4 (42.9–55.1)	
		n = 154	
SF-10 PHS, mean (SD)/median (IQR)	NA	NA	38.0 (14.5)
			42.0 (32.5–48.4)

ADerm-IS, Atopic Dermatitis Impact Scale; ADerm-SS, Atopic Dermatitis Symptom Scale; ADerm-SS TSS-7, Atopic Dermatitis Symptom Scale Total Symptom Score-7; EASI, Eczema Area and Severity Index; IQR, Interquartile Range; POEM, Patient Oriented Eczema Measurement; SCORAD, SCORing Atopic Dermatitis; SF-12 PCS, 12-item Short-Form Health Survey Physical Component Summary (adults); SF-10 PHS, 10-item Short-Form Health Survey Physical Health Score (adolescents); vIGA-AD, Validated Investigator Global Assessment for Atopic Dermatitis.

The mean vIGA-AD was 2.9 (adults, 3.0; adolescents, 2.5) and the mean EASI score was 17.0 (adults, 17.7; adolescents, 12.4). Overall, 3.9% of patients had clear skin (EASI = 0) and 26.7% had severe AD (EASI 23–72).

The mean ADerm-SS Skin Pain was 4.4, indicating moderate disease (Table 2). The mean ADerm-SS TSS-7 and TSS-11 scores (34.2 and 49.2, respectively) showed similarly moderate symptom burden (Table 2).

A total of 169 patients reported a frequency of flares. Over the previous 6 months, 0, 1 to 2, 3 to 4, 5 to 6, and > 6 flares were reported by 8.3%, 27.2%, 31.1%, 11.7%, and 15.6% of patients, respectively. On average, flares lasted 15.2 days (adults, 15.9 days; adolescents, 11.1 days).

Borderline abnormal to abnormal anxiety (HADS-A ≥ 8) and depression (HADS-D ≥8) were reported by 55.0% and 33.3% of patients in the total population; a slightly higher rate of anxiety was reported among adolescents (65.2%) versus adults (53.5%; Table 3). The mean SF-12 mental component summary score was 41.8 for adults and the mean SF-10 psychosocial component summary score was 44.6 for adolescents (Table 3). A mean work productivity loss of 35.9% was observed among employed adults (Table 3). Similar effects of AD were reflected in the ADerm-IS Emotional State scores (mean, 14.3) and ADerm-IS Daily Activities scores (mean, 14.6), indicating moderate disease (Table 3). The mean number of healthcare or acute care visits during the previous 6 months was 7.6; this was similar for adults (7.7) and adolescents (7.1) (Table 3). Mean monthly healthcare-related expenses and costs of everyday necessities related to AD (converted to 2021 US Dollars [USD]) were 103.3 USD in the total population (adults, 101.8 USD; adolescents 113.4 USD).

**Table 3:** Psychosocial-economic burden of AD.

	Total population (n = 180)	Adults (n = 157)	Adolescents (n = 23)
HADS Anxiety subscale, mean (SD)/median (IQR)	8.4 (4.7)	8.3 (4.7)	9.0 (4.4)
	8.0 (5.0–12.0)	8.0 (5.0–12.0)	10.0 (6.0–12.0)
HADS Anxiety subscale ≥8, n (%)	99 (55.0)	84 (53.5)	15 (65.2)
HADS Depression subscale, mean (SD)/median (IQR)	6.0 (3.8)	5.9 (3.9)	6.1 (3.5)
	6.0 (3.0–8.0)	6.0 (3.0–8.0)	6.0 (3.0–8.0)
HADS Depression subscale ≥8, n (%)	60 (33.3)	52 (33.1)	8 (34.8)
SF-12 MCS, mean (SD)/median (IQR)	NA	41.8 (11.1)	NA
		40.4 (33.9–51.0)	
		n = 154	
SF-10 PSS, mean (SD)/median (IQR)	NA	NA	44.6 (9.2)
			45.3 (36.4–51.6)
ADerm-IS Daily Activities, mean (SD)/median (IQR)	14.6 (12.5)	15.6 (12.5)	8.0 (10.5)
	11.0 (3.0–24.0)	14.0 (4.0–25.0)	3.0 (2.0–14.0)
	n = 178	n = 155	
ADerm-IS Emotional State, mean (SD)/median (IQR)	14.3 (10.9)	15.3 (10.8)	7.1 (9.3)
	15.0 (3.0–25.0)	18.0 (4.0–25.0)	3.0 (0.0–12.0)
WPAI-AD employed, n (%)	83 (46.1)	79 (50.3)	4 (17.4)
Absenteeism, mean (SD)/median (IQR), %	12.1 (21.9)	12.0 (22.2)	14.2 (18.9)
	0.0 (0.0–16.7)	0.0 (0.0–16.7)	8.3 (0.0–28.3)
	n = 75	n = 71	n = 4
Presenteeism, mean (SD)/median (IQR), %	31.2 (28.0)	32.0 (28.0)	15.0 (23.8)
	25.0 (10.0–50.0)	30.0 (10.0–50.0)	5.0 (0.0–30.0)
	n = 78	n = 74	n = 4
Overall work productivity impairment, mean (SD)/median (IQR), %	35.3 (28.7)	35.9 (28.8)	26.1 (30.5)
	32.3 (10.0–58.3)	32.3 (10.0–58.3)	23.0 (0.0–52.2)
	n = 73	n = 69	n = 4
Hours missed from work, mean (SD)/median (IQR)	7.0 (22.2)	7.3 (22.8)	1.3 (1.5)
	0.0 (0.0–4.0)	0.0 (0.0–4.0)	1.0 (0.0–2.5)
	n = 78	n = 74	n = 4
Activity impairment, mean (SD)/median (IQR), %	39.8 (34.8)	42.2 (35.2)	23.9 (23 (26.9)
	30.0 (10.0–70.0)	30.0 (10.0–70.0)	20.0 (0.0–40.0)
Healthcare resource utilization			
Number of healthcare or acute care visits in previous 6-months, mean (SD)	7.6 (9.4)	7.7 (9.7)	7.1 (7.1)
	n = 123	n = 108	n = 15
Out-of-pocket expenses			
Total monthly healthcare-related expenses and costs of everyday necessities related to AD, mean (SD), USD	103.3 (132.1)	101.8 (137.8)	113.4 (87.5)
	n = 168	n = 146	n = 22

AD, Atopic Dermatitis; ADerm-IS, Atopic Dermatitis Impact Scale; ADerm-SS, Atopic Dermatitis Symptom Scale; HADS, Hospital Anxiety and Depression Scale; IQR, Interquartile Range; SD, Standard Deviation; SF-12 MCS, 12-item Short-Form Health Survey Mental Component Summary (adults); SF-10 PSS, 10-item Short-Form Health Survey Psychosocial Summary Score (adolescents); USD, US Dollars; WPAI-AD, Work Productivity and Activity Impairment-Atopic Dermatitis.

### Subgroup analyses

In a subgroup analysis by current systemic therapy use (yes, n = 118; no, n = 62), no significant differences in primary or secondary endpoints were observed between groups (Table 4). The only exceptions were a slightly higher mean SF-12 mental component summary score (worse mental health) and a greater proportion of patients with EASI ≥ 16.0 among systemic therapy users versus non-systemic therapy users (Table 4). Substantial disease burden was observed among patients who were and who were not currently receiving systemic therapy, as based on mean WP-NRS (6.1 and 6.1), mean DLQI (11.8 and 10.7), mean EASI (18.1 and 14.7), mean SCORAD (53.2 and 51.3), and mean number of flares in the past 6 months (4.8 and 5.3, respectively) (Table 4).

**Table 4:** Clinical, psychosocial, and economic burden of AD by systemic therapy use.

	Current Use of Systemic Therapy					
	Yes			No	p-value	
	Any systemic (n = 118)	Dupilumab (n = 29)	Systemic other than dupilumab (n = 89)	No systemic therapy (n = 62)	Systemic vs. no systemic	Dupilumab vs. other systemic
Primary endpoints						
WP-NRS, mean (SD)/median (IQR)	6.1 (.1)	4.2 (3.3)	6.7 (2.7)	6.1 (3.0)	0.987	**<0.001**
	7.0 (4.0–8.0)	4.0 (1.0–7.0)	7.0 (5.0–9.0)	7.0 (3.0–8.0)		
WP-NRS 0–1, n (%)	14 (11.9)	9 (31.0)	5 (5.6)	7 (11.3)	0.909	**<0.001**
WP-NRS ≥ 4, n (%)	90 (76.3)	16 (55.2)	74 (83.1)	46 (74.2)	0.758	**0.002**
DLQI, mean (SD)/median (IQR)	11.8 (8.0)	9.6 (8.6)	12.6 (7.6)	10.7 (7.3)	0.451	0.055
	11.0 (5.0–18.0)	8.0 (2.0–15.0)	12.0 (5.0–19.0)	9.0 (4.0–15.0)		
	n = 104	n = 26	n = 78	n = 58		
CDLQI, mean (SD)/median (IQR)	8.3 (7.3)	5.3 (8.4)	9.6 (7.1)	7.8 (6.2)	0.943	0.305
	7.0 (2.0–14.0)	1.0 (0.0–15.0)	9.0 (3.0–14.0)	8.0 (3.0–13.0)		
	n = 10	n = 3	n = 7	n = 4		
Clinical outcomes						
EASI, mean (SD)/median (IQR)	18.2 (12.6)	12.2 (10.7)	20.1 (12.7)	14.7 (8.4)	0.131	**0.002**
	17.0 (8.6–26.4)	10.7 (1.9–17.2)	18.3 (11.0–26.8)	13.2 (9.0–20.4)		
EASI ≥ 7.1, n (%)	94 (79.7)	19 (65.5)	75 (84.3)	55 (88.7)	0.127	**0.029**
EASI ≥ 16.0, n (%)	62 (52.5)	10 (34.5)	52 (58.4)	23 (37.1)	**0.049**	**0.025**
SCORAD, mean (SD)/median (IQR)	53.2 (23.1)	‒	‒	51.3 (15.0)	0.236	‒
	57.6 (40.4–70.6)			52.0 (41.0–61.7)		
	n = 117			n = 62		
SCORAD ≥ 25, n (%)	103 (88.0)	‒	‒	59 (95.2)	0.122	‒
vIGA-AD categories, n (%)					0.055	‒
Clear (0)	5 (4.2)	‒	‒	1 (1.6)		
Almost clear (1)	7 (5.9)	‒	‒	5 (8.1)		
Mild (2)	12 (10.2)	‒	‒	5 (8.1)		
Moderate (3)	56 (47.5)	‒	‒	42 (67.7)		
Severe (4)	38 (32.2)	‒	‒	9 (14.5)		
Body surface area, %, mean (SD)/median (IQR)	28.1 (22.8)	‒	‒	23.4 (17.8)	0.283	‒
	25.0 (10.0–40.0)			20.0 (8.0–38.0)		
Number of flares in the past 6 months, mean (SD)/median (IQR)	4.8 (6.8)	4.3 (5.8)	5.0 (7.1)	5.3 (7.5)	0.433	0.167
	3.0 (2.0–6.0)	3.0 (1.0–4.0)	3.0 (2.0–6.0)	4.0 (2.0–6.0)		
	n = 110	n = 28	n = 82	n = 59		
Number of flares categories, n (%)					0.358	0.253
0	9 (8.2)	4 (14.3)	5 (6.1)	6 (10.2)		
1–2	35 (31.8)	9 (32.1)	26 (31.7)	14 (23.7)		
3–4	36 (32.7)	9 (32.1)	27 (32.9)	20 (33.9)		
5–6	10 (9.1)	0	10 (12.2)	11 (18.6)		
> 6	20 (18.2)	6 (21.4)	14 (17.1)	8 (13.6)		
Inadequately controlled AD, n (%)	33 (28.0)	‒	‒	12 (19.4)	0.205	‒
Average hours of sleep per night in the past week, mean (SD)/median (IQR)	6.1 (1.9)	‒	‒	6.1 (1.9)	0.899	‒
	6.0 (5.0–8.0)			6.0 (5.0–7.0)		
Psychosocial outcomes						
HADS Anxiety subscale ≥8, n (%)	66 (55.9)	‒	‒	33 (53.2)	0.729	‒
HADS Depression subscale ≥8, n (%)	41 (34.7)	‒	‒	19 (30.6)	0.579	‒
SF-12 MCS, mean (SD)/median (IQR)	40.4 (10.9)	‒	‒	44.2 (11.2)	**0.046**	‒
	38.7 (33.2–49.4)			45.2 (35.7–53.4)		
	n = 98			n = 56		
Work Productivity and Activity Impairment						
Absenteeism, mean (SD)/median (IQR), %	11.9 (22.4)	14.6 (21.8)	11.2 (22.8)	12.3 (21.4)	0.519	0.171
	0.0 (0.0–16.7)	6.2 (0.0–19.1)	0.0 (0.0–16.7)	4.8 (0.0–16.7)		
	n = 48	n = 10	n = 38	n = 27		
Presenteeism, mean (SD)/median (IQR), %	30.6 (27.1)	41.0 (32.5)	27.9 (25.4)	32.1 (29.8)	0.963	0.215
	20.0 (10.0–50.0)	30.0 (10.0–60.0)	20.0 (0.0–50.0)	30.0 (0.0–50.0)		
	n = 49	n = 10	n = 39	n = 29		
Overall work productivity impairment, mean (SD)/median (IQR), %	35.8 (28.3)	45.1 (32.3)	33.2 (27.0)	34.5 (30.1)	0.876	0.246
	32.3 (10.0–55.0)	42.9 (12.5–67.7)	31.2 (10.0–50.0)	29.2 (4.8–58.5)		
	n = 47	n = 10	n = 37	n = 26		
Activity impairment, mean (SD)/median (IQR), %	41.2 (36.5)	30.0 (37.8)	44.8 (35.6)	37.3 (31.2)	0.679	**0.032**
	30.0 (10.0–70.0)	10.0 (0.0–60.0)	40.0 (10.0–70.0)	30.0 (10.0–60.0)		
Hours missed from work, mean (SD)/median (IQR)	7.4 (25.4)	5.5 (9.2)	7.9 (28.4)	6.2 (15.4)	0.262	0.152
	0.0 (0.0–3.0)	2.0 (0.0–7.0)	0.0 (0.0–2.0)	2.0 (0.0–5.0)		
	n = 50	n = 11	n = 39	n = 28		
Healthcare resource utilization						
Number of acute care visits in the previous 6-months, mean (SD)/median (IQR)	0.4 (2.8)	‒	‒	0	0.053	‒
	0.0 (0.0–0.0)			0.0 (0.0–0.0)		
	n = 84			n = 37		
Number of healthcare visits in the previous 6-months, mean (SD)/median (IQR)	7.1 (7.6)	6.5 (5.7)	7.3 (8.2)	7.8 (12.2)	0.987	0.622
	4.0 (2.0–8.0)	6.0 (3.0–8.0)	4.0 (2.0–8.0)	4.5 (2.0–8.0)		
	n = 87	n = 21	n = 66	n = 36		
Monthly healthcare-related expenses and costs due to AD, mean (SD)/median (IQR), USD	108.4 (146.2)	113.2 (134.9)	106.8 (150.7)	93.6 (100.6)	0.778	0.471
	64.5 (33.4–120.0)	74.4 (41.1–109.2)	60.0 (33.0–126.0)	59.5 (33.6–117.0)		
	n = 110	n = 28	n = 82	n = 58		

AD, Atopic Dermatitis; CDLQI, Children's Dermatology Life Quality Index; DLQI, Dermatology Life Quality Index; EASI, Eczema Area and Severity Index; HADS, Hospital Anxiety and Depression Scale; IQR, Interquartile Range; SCORAD, SCORing Atopic Dermatitis; SF-12 MCS, 12-item Short-Form Health Survey Mental Component Summary; USD, US Dollars; vIGA-AD, Validated Investigator Global Assessment for Atopic Dermatitis; WP-NRS, Worst Pruritus Numeric Rating Scale.

For comparison of dupilumab (monotherapy or combination, n = 29) versus other systemic therapy (n = 89), patients receiving dupilumab generally had lower disease severity and activity impairment, but differences were only significant for mean EASI (p = 0.002), mean WP-NRS (p < 0.001), and activity impairment (p = 0.032) and for proportions of patients with EASI ≥ 7.1 (p = 0.029), EASI ≥ 16.0 (p = 0.025), WP-NRS 0–1 (p < 0.001) and WP-NRS ≥ 4 (p = 0.002; Table 4). Substantial disease burden was still observed among patients receiving dupilumab based on mean WP-NRS (4.2), mean DLQI (9.6), mean EASI (12.2), and mean number of flares in the last 6 months (4.3) (Table 4).

**Fig. 3:**
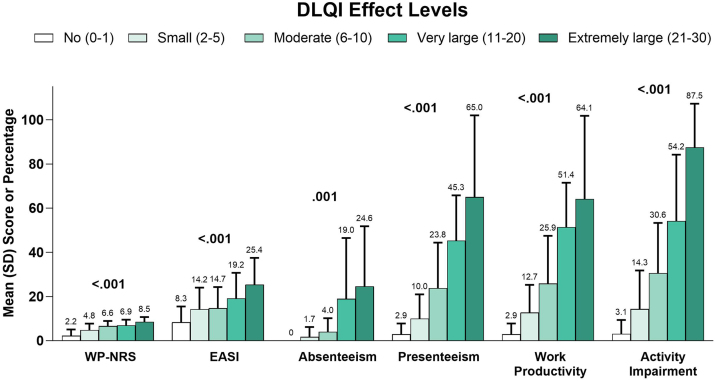
Clinical outcomes and economic burden of AD by DLQI. AD, Atopic Dermatitis; DLQI, Dermatology Life Quality Index; EASI, Eczema Area and Severity Index; WP-NRS, Worst Pruritus Numeric Rating Scale.

**Fig. 4:**
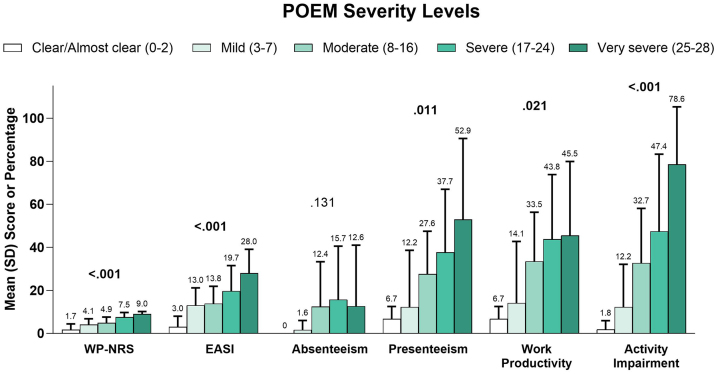
Clinical outcomes and economic burden of AD by POEM. AD, Atopic Dermatitis; EASI, Eczema Area and Severity Index; POEM, Patient Oriented Eczema Measurement; WP-NRS, Worst Pruritus Numeric Rating Scale.

**Fig. 5:**
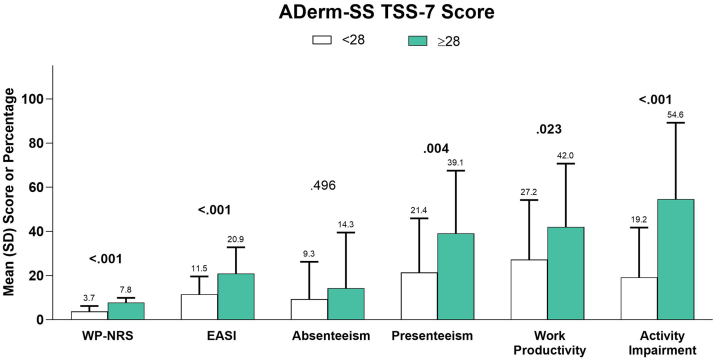
Clinical outcomes and economic burden of AD by ADerm-SS TSS-7. AD, Atopic Dermatitis; ADerm-SS TSS-7, Atopic Dermatitis Symptom Scale Total Symptom Score-7; EASI, Eczema Area and Severity Index; WP-NRS, Worst Pruritus Numeric Rating Scale.

In a subgroup analysis by EASI severity, overall work productivity impairment, activity impairment, and overall number of acute care visits were significantly associated with increasing disease severity (Table 5). Similarly, higher DLQI (Fig. 3), more severe POEM (Fig. 4), and greater ADerm-SS TSS-7 (Fig. 5) were associated with significantly greater disease severity, itch severity, and impairments in work productivity and activity.

**Table 5:** Psychosocial and economic burden of AD by EASI disease severity level.

	EASI Severity Levels				
	Clear (n = 7)	Mild (n = 18)	Moderate (n = 107)	Severe (n = 48)	p-value
Psychosocial outcomes					
HADS Anxiety subscale ≥ 8, n (%)	4 (57.1)	7 (38.9)	57 (53.3)	31 (64.6)	0.298
HADS Depression subscale ≥ 8, n (%)	2 (28.6)	6 (33.3)	28 (26.2)	24 (50.0)	0.059
SF-12 MCS, mean (SD)/median (IQR)	42.3 (15.3)	44.3 (10.0)	43.0 (10.3)	38.1 (12.3)	0.072
	43.4 (29.7–54.9)	41.6 (37.6–49.5)	43.4 (34.7–51.4)	35.7 (29.7–50.1)	
	n = 4	n = 14	n = 94	n = 42	
Work Productivity and Activity Impairment					
Absenteeism, mean (SD)/median (IQR), %	12.5 (NA)	1.3 (2.3)	11.0 (20.7)	20.9 (28.6)	0.127
	12.5 (12.5–12.5)	0.0 (0.0–2.4)	0.0 (0.0–12.7)	10.8 (0.0–28.8)	
	n = 1	n = 8	n = 50	n = 16	
Presenteeism, mean (SD)/median (IQR), %	50.0 (NA)	18.9 (19.7)	29.2 (27.9)	43.1 (29.8)	0.121
	50.0 (50.0–50.0)	20.0 (0.0–20.0)	20.0 (5.0–45.0)	45.0 (20.0–60.0)	
	n = 1	n = 9	n = 52	n = 16	
Overall work productivity impairment, mean (SD)/median (IQR), %	56.3 (NA)	16.0 (17.3)	32.9 (28.8)	52.1 (26.1)	**0.029**
	56.3 (56.3–56.3)	15.0 (0.0–24.0)	30.0 (10.0–55.0)	50.0 (31.2–70.0)	
	n = 1	n = 8	n = 49	n = 15	
Activity impairment, mean (SD)/median (IQR), %	12.9 (26.3)	16.1 (18.5)	34.1 (31.4)	65.4 (33.2)	**<0.001**
	0.0 (0.0–20.0)	10.0 (0.0–30.0)	30.0 (10.0–60.0)	75.0 (35.0–100.0)	
Hours missed from work, mean (SD)/median (IQR)	6.0 (NA)	0.4 (0.9)0.0 (0.0–0.0)	7.3 (25.6)	9.6 (16.4)	0.119
	6.0 (6.0–6.0)	n = 9	0.0 (0.0–4.0)	2.0 (0.0–12.0)	
	n = 1		n = 52	n = 16	
Healthcare resource utilization					
Number of acute care visits in the previous 6-months, mean (SD)/median (IQR)	0	0	0 (0.17)	1.1 (4.5)	**0.037**
	0.0 (0.0–0.0)	0.0 (0.0–0.0)	0.0 (0.0–0.0)	0.0 (0.0–0.0)	
	n = 5	n = 14	n = 71	n = 31	
Number of healthcare visits in the previous 6 months, mean (SD)/median (IQR)	6.5 (7.8)	13.6 (12.1)	7.1 (9.9)	6.4 (5.9)	0.574
	6.5 (1.0–12.0)	9.0 (3.0–27.0)	4.0 (2.0–7.0)	4.0 (3.0–8.0)	
	n = 2	n = 9	n = 75	n = 37	
Monthly healthcare-related expenses and costs due to AD, mean (SD)/median (IQR), USD	94.0 (80.1)	99.4 (63.6)	89.7 (106.6)	135.0 (191.3)	0.288
	72.0 (48.0–90.0)	66.0 (49.5–144.0)	54.5 (31.2–110.4)	67.5 (30.2–144.0)	
		n = 17	n = 98	n = 46	

AD, Atopic Dermatitis; EASI, Eczema Area and Severity Index; HADS, Hospital Anxiety and Depression Scale; IQR, Interquartile Range; SD, Standard Deviation; SF-12 MCS, 12-item Short-Form Health Survey Mental Component Summary; USD, US Dollars.

## Discussion

This analysis of 180 adults and adolescents with AD from 3 Latin American countries demonstrated considerable clinical, psychosocial, and economic burden of disease. More than half of the adult patients (57%) and 35% of adolescent patients reported severe itch. A very large or extremely large effect on QoL was reported by 50% of adults and 43% of adolescents. In addition, anxiety, depression, and sleep problems were prevalent, with 54% of adults reporting HADS-A ≥ 8 (65% of adolescents), 33% reporting HADS-D ≥ 8 (35% of adolescents), and 53% reporting sleep problems occurring somewhat to very much (17% of adolescents).

Although all patients received treatment and two-thirds received systemic therapies, moderate to severe disease was reported by 90% and 86% of patients based on SCORAD and EASI scores, respectively, suggesting inadequately controlled disease. In a subgroup analysis by systemic therapy use, no difference in disease burden was observed between patients receiving systemic therapy versus patients not receiving systemic therapy; disease burden (including number of flares, QoL impact, and economic burden) remained high in both groups. However, among patients receiving the biologic therapy dupilumab, a trend for lower disease severity, better QoL, and lower activity impairment was observed compared with patients receiving other conventional systemic therapies or patients not receiving systemic therapy. Nevertheless, a substantial disease burden was still observed among patients receiving dupilumab, indicating a remaining unmet need for effective AD therapies.

Overall, the results in the Latin American population from MEASURE-AD indicated that a considerable disease burden still exists among patients with AD, regardless of current treatment, including systemic therapy.

These results are also in line with previous studies from Latin America. In a retrospective, registry-based study conducted in Brazil between 2016 and 2017, 87% (85/98) of patients had moderate to severe disease as assessed by SCORAD and 75% (24/32) as assessed by EASI.^17^ The use of oral corticosteroids (33% [61/187]) was slightly higher in that population than in the MEASURE-AD Latin America population (23% [41/180]).^17^ In another observational study from Colombia enrolling patients between 2019 and 2020, 76% (SCORAD) and 61% (EASI) of patients had moderate to severe disease with 63% (133/212) using oral corticosteroids and 7% (14/212) using dupilumab (vs. 23% [41/180] and 16% [29/180], respectively, in MEASURE-AD Latin America).^16^ Similar results have been demonstrated in other studies from Latin America.^15^, ^23^ Furthermore, psychological, social-functioning, and economic effects are also relevant among patients from Latin America.^15^, ^18^ In a web-based survey involving 1650 adult and pediatric patients with AD in Argentina, 86.5% of patients reported a negative impact on QoL, with frustration, anger, mood alternations, stress, sleep and routine alterations, pain, and economic impact being among the highest ranked domains.^18^ Topical treatments were frequently used, including by 60% of patients receiving topical corticosteroids; however, 21.7% of patients reported treatment satisfaction as moderate, and 40.5% were dissatisfied with their treatment regimen. Another important finding from the study was a delay in diagnosis, which was more evident in the provinces away from Buenos Aires, and a lack of knowledge about the diagnosis of the disease among specialists.^18^

Results from studies in children and adolescents with AD also show a high impact of disease. In the international PEDISTAD study of 732 children (with 23% from Latin America), a significant impact of moderate to severe AD on itch, sleep, QoL, and family was demonstrated in children and their caregivers. This might have been explained by the low use of systemic therapies (23%).^24^ Similarly, a study of 50 children and adolescents with AD from Brazil demonstrated a moderate to high negative effect of AD on QoL in 72% of patients and 74% of families.^25^ These results are consistent with the findings of the MEASURE-AD study, which demonstrated considerable disease burden (including itch) and sleep and QoL impairment among Latin American adolescents, of whom only 22% used systemic treatment continuously over the previous 12 months.

A slightly higher disease burden was reported in the Latin American population versus the global MEASURE-AD population, in which severe itch was reported by 42% of patients, a very large or extremely large effect on QoL was reported by 46% of adults and 32% of adolescents, and moderate to severe disease was reported by 69% (EASI ≥ 6) and 76% (SCORAD ≥ 25) of patients.^20^

Interestingly, the time to first systemic therapy was longer in the MEASURE-AD global (17 years) versus Latin American (10 years) population, which may indicate that the Latin American patients with AD sought treatment later in the disease course and escalated to systemic therapy more rapidly than the global population. Although the use of systemic therapies was slightly higher in the Latin American versus global population (66% vs. 56%), the use of biologic therapy (dupilumab) among systemic therapy users was considerably lower in Latin America (25% vs. 56%, respectively), which could explain the higher disease burden. In contrast, the use of systemic corticosteroids (35% vs. 18%) and methotrexate (33% vs. 16%) among systemic therapy users was higher in the Latin American versus the global population. Among all adult patients enrolled in MEASURE-AD (n = 1434), the use of biologic therapy (dupilumab) was much lower in Latin America (15% [24/157]) compared with other geographic regions, such as Saudi Arabia, Kuwait, and United Arab Emirates (62% [26/42]), Italy (57% [67/118]), Germany (46% [96/210]), Canada (44% [88/200]), Switzerland and Austria (46% [43/94]), and Spain (42% [38/91]). In Latin America, the cost of systemic medication affects treatment choices in addition to access to treatment. Both methotrexate and oral corticosteroids are less expensive and easier to administer, which may explain their high use versus dupilumab. Of note, the COVID-19 pandemic was ongoing at the time of the study, which could have affected access to dupilumab and lowered its use. Overall, these findings may be explained by market access differences across countries and indicate that not all patients from Latin America have access to systemic medications and remain undertreated.^26^

Of note, dupilumab was the only biological therapy approved for AD at the time of MEASURE-AD study. The number of treatment options for AD has since increased with the approval of Janus kinase inhibitors (baricitinib, upadacitinib, and abrocitinib) and biologics (such as tralokinumab) in some countries. Thus, future studies are needed to assess the effect of the approval of these therapies on AD disease burden.

The strengths of this study included the reporting of a wide variety of both physician- and patient-reported outcomes, including QoL. Furthermore, this was the largest study assessing the multidimensional burden of AD (clinical, psychological, socioeconomic) and the impact of available systemic treatments on disease burden in Latin America. Limitations of this study included relying on patient self-reported measures and recall during a single office visit and that only patients who were receiving or were candidates for systemic therapy were included. Furthermore, dupilumab was the only approved biological therapy at the time of the study, which was conducted before Janus kinase inhibitors were available. In addition, local regulatory approval status and market access/reimbursement policies varied across countries and some patients had limited access to innovative treatments.

## Conclusion

Patients with moderate to severe AD in Brazil, Mexico, and Argentina continue to experience substantial multidimensional disease burden and uncontrolled disease. Although most patients used topical treatments, two-thirds were receiving systemic treatments (mainly corticosteroids or methotrexate) either alone or in combination. Future studies need to look at the effect of newer and more effective therapies on the burden of disease. Overall, a significant unmet need remains for effective treatments to improve patients’ psychosocial and clinical outcomes and reduce the economic burden of AD.

## Study conducted at the 14 study sites in Argentina, Brazil, and México

*Argentina*

- Fundación Cidea Allergy and Respiratory Research Unit, Buenos Aires, Argentina
- Hospital Italiano de Buenos Aires, Ciudad Autónoma de Buenos Aires, Argentina
- Instituto de Neumonologia y Dermatologia, Hospital Alemán, Ciudad Autónoma de Buenos Aires, Argentina
- Psoriahue Medicina Interdisciplinaria, Ciudad Autónoma de Buenos Aires, Argentina

*Brazil*

- Centro Universitário Saúde ABC, Santo André, SP, Brazil
- Clínica de Alergía, Sorocaba, SP, Brazil
- Pontifícia Universidade Católica do Paraná, Curitiba, PR, Brazil
- Santa Casa de Misericordia de Porto Alegre, Porto Alegre, RS, Brazil
- University of Rio Grande do Sul - Hospital de Clínicas de Porto Alegre, Porto Alegre, RS, Brazil
- University of São Paulo School of Medicine, São Paulo, SP, Brazil

México

- GA^2^LEN Atopic Dermatitis Center of Reference and Excellence, Secretaria de la Defensa Nacional, Ciudad de México, México
- Grupo Clínico CATEI (Centro de Atención en Enfermedades Inflamatorias) Sociedad Civil, Guadalajara, Jal, México
- Instituto Nacional de Ciencias Médicas y Nutrición Salvador Zubirán, Ciudad de México, México
- NEKI Servicios Médicos, Vicente Guerreo, Toluca, Méx, México

## Financial support

AbbVie Inc. funded the studies and participated in the study design, research, analyses, data collection and interpretation, reviewing, and approval of the publication. No honoraria or payments were made for authorship. All authors had access to relevant data and participated in the drafting, review, and approval of this publication.

## Authors’ contributions

Catalina Rincón Pérez: Approval of the final version of the manuscript; critical literature review; data collection, analysis, and interpretation; intellectual participation in propaedeutic and/or therapeutic management of studied cases; effective participation in research orientation; analysis and interpretation of data; manuscript critical review; preparation and writing of the manuscript.

Valeria Aoki: Approval of the final version of the manuscript; critical literature review; data collection, analysis, and interpretation; intellectual participation in propaedeutic and/or therapeutic management of studied cases; effective participation in research orientation; analysis and interpretation of data; manuscript critical review; preparation and writing of the manuscript.

Roberta F. Criado: Approval of the final version of the manuscript; data collection, analysis, and interpretation; intellectual participation in propaedeutic and/or therapeutic management of studied cases; effective participation in research orientation; analysis and interpretation of data; manuscript critical review; preparation and writing of the manuscript.

Martti Antila: Approval of the final version of the manuscript; critical literature review; data collection, analysis, and interpretation; intellectual participation in propaedeutic and/or therapeutic management of studied cases; effective participation in research orientation; analysis and interpretation of data; manuscript critical review; preparation and writing of the manuscript.

Maria Valeria Angles: Approval of the final version of the manuscript; data collection, analysis, and interpretation; intellectual participation in propaedeutic and/or therapeutic management of studied cases; effective participation in research orientation; analysis and interpretation of data; manuscript critical review; preparation and writing of the manuscript.

Tania Ferreira Cestari: Approval of the final version of the manuscript; data collection, analysis, and interpretation; intellectual participation in propaedeutic and/or therapeutic management of studied cases; effective participation in research orientation; analysis and interpretation of data; manuscript critical review; preparation and writing of the manuscript.

Delfina Guadalupe Villanueva Quintero: Approval of the final version of the manuscript; data collection, analysis, and interpretation; intellectual participation in propaedeutic and/or therapeutic management of studied cases; effective participation in research orientation; analysis and interpretation of data; manuscript critical review; preparation and writing of the manuscript.

Gabriel Magariños: Approval of the final version of the manuscript; data collection, analysis, and interpretation; intellectual participation in propaedeutic and/or therapeutic management of studied cases; effective participation in research orientation; analysis and interpretation of data; manuscript critical review; preparation and writing of the manuscript.

Carla Castro: Approval of the final version of the manuscript; data collection, analysis, and interpretation; intellectual participation in propaedeutic and/or therapeutic management of studied cases; effective participation in research orientation; analysis and interpretation of data; manuscript critical review; preparation and writing of the manuscript.

Adriana López Tello-Santillán: Approval of the final version of the manuscript; data collection, analysis, and interpretation; intellectual participation in propaedeutic and/or therapeutic management of studied cases; effective participation in research orientation; analysis and interpretation of data; manuscript critical review; preparation and writing of the manuscript.

Magda Weber: Approval of the final version of the manuscript; data collection, analysis, and interpretation; intellectual participation in propaedeutic and/or therapeutic management of studied cases; effective participation in research orientation; analysis and interpretation of data; manuscript critical review; preparation and writing of the manuscript.

Daniel Lorenzini: Approval of the final version of the manuscript; data collection, analysis, and interpretation; intellectual participation in propaedeutic and/or therapeutic management of studied cases; effective participation in research orientation; analysis and interpretation of data; manuscript critical review; preparation and writing of the manuscript.

Caio Cesar Silva de Castro: Approval of the final version of the manuscript; data collection, analysis, and interpretation; intellectual participation in propaedeutic and/or therapeutic management of studied cases; effective participation in research orientation, analysis and interpretation of data; manuscript critical review; preparation and writing of the manuscript.

Jorge Maspero: Approval of the final version of the manuscript; critical literature review; data collection, analysis, and interpretation; intellectual participation in propaedeutic and/or therapeutic management of studied cases; effective participation in research orientation; analysis and interpretation of data; manuscript critical review; preparation and writing of the manuscript.

Linda García-Hidalgo: Approval of the final version of the manuscript; data collection, analysis, and interpretation; intellectual participation in propaedeutic and/or therapeutic management of studied cases; effective participation in research orientation; analysis and interpretation of data; manuscript critical review; preparation and writing of the manuscript.

Limei Zhou: Approval of the final version of the manuscript; data collection, analysis, and interpretation; analysis and interpretation of data; manuscript critical review; preparation and writing of the manuscript; statistical analysis; study conception and planning.

Shereen Hammad: Approval of the final version of the manuscript; critical literature review; data collection, analysis, and interpretation; analysis and interpretation of data; manuscript critical review; preparation and writing of the manuscript; study conception and planning.

Lucila de Campos: Approval of the final version of the manuscript; critical literature review; data collection, analysis, and interpretation; analysis and interpretation of data; manuscript critical review; preparation and writing of the manuscript; study conception and planning.

Tatiane Cristina Rodrigues: Approval of the final version of the manuscript; critical literature review; data collection, analysis, and interpretation; analysis and interpretation of data; manuscript critical review; preparation and writing of the manuscript; study conception and planning.

Carolina Arzelán: Approval of the final version of the manuscript; critical literature review; data collection, analysis, and interpretation; analysis and interpretation of data; manuscript critical review; preparation and writing of the manuscript; study conception and planning.

Paula C. Luna: Approval of the final version of the manuscript; data collection, analysis, and interpretation; intellectual participation in propaedeutic and/or therapeutic management of studied cases; effective participation in research orientation; analysis and interpretation of data; manuscript critical review; preparation and writing of the manuscript.

## Conflicts of interest

Catalina Rincón Pérez has been an investigator, speaker, and/or advisor for AbbVie and a speaker and/or advisor for Janssen, Leo, Lilly, Novartis, Pfizer, and Sanofi Genzyme.

Valeria Aoki has received research grants as an investigator from Lilly and Sanofi Laboratories (the funds were administered by her institution) and served as an advisor/speaker for AbbVie, LEO Pharma, and Pfizer.

Roberta F. Criado has served as an advisor and/or speaker for AbbVie, Mantecorp, Novartis, and Sanofi.

Martti Antila has served as a speaker/consultant for Abbott, AbbVie, Aché, AstraZeneca, Chiesi, Eurofarma, IPI ASAC, and Sanofi and received research support from AbbVie, AstraZeneca, EMS, Eurofarma, GSK, Humanigen, Janssen, Novartis, Sanofi, and Veru.

Maria Valeria Angles has received honoraria or fees for serving on advisory boards, as a speaker, and as a consultant; grants as an investigator from AbbVie, L'Oréal, Pfizer, Raffo, and Sanofi; and grants/research support (paid to the institution) from or participated in clinical trials for AbbVie and Sanofi.

Tania Ferreira Cestari has received research grants as an investigator from AbbVie (including for the MEASURE-AD study), Janssen Cilag, Lilly, Pfizer, and Vichy Laboratories; the funds were administered by her institution (Hospital de Clinicas de Porto Alegre).

Delfina Guadalupe Villanueva Quintero has served as a speaker, advisor, and principal investigator for AbbVie, Amgen, BI, BMS, Janssen, Lilly, Novartis, Sanofi, and Teva.

Gabriel Magariños declares consultancy fees from AbbVie, BI, BMS, Janssen, Lilly, Novartis, Pfizer, and Sanofi, and research grants from AbbVie, BI, BMS, Janssen, Lilly, Merck, MSD, Novartis, and Pfizer.

Carla Castro has received consultancy fees and/or research grants from AbbVie, Amgen, BI, Biogen, Galderma, Isdin, Janssen, Knight, Lilly, L'Oréal, Merck, Novartis, Pfizer, and Sanofi.

Adriana López Tello-Santillán has served as a speaker, investigator, and advisor for AbbVie, Janssen, LEO, Lilly, Novartis, Pfizer, and UCB.

Magda Weber has been an investigator, speaker, and/or advisor for AbbVie and has received funds from ISCMPA.

Daniel Lorenzini has been an investigator, speaker, and/or advisor for AbbVie, Galderma, LEO, Lilly, Pfizer, and Sanofi-Genzyme.

Caio Cesar Silva de Castro has served as a speaker or consultant for AbbVie, Aché, Janssen, Knight, LEO, Novartis, Sanofi, and Sun Pharma.

Jorge Maspero has been an investigator for, received grants or speaker fees from, or has been an advisor for AbbVie, AstraZeneca, GSK, Inmunotek, Menarini, MSD, Novartis, Sanofi-Genzyme, and Uriach.

Linda García-Hidalgo has served as a speaker and advisor for AbbVie, Eucerin, Janssen, Novartis, Novo Nordisk, and Sanofi.

Limei Zhou, Shereen Hammad, Lucila de Campos, Tatiane Cristina Rodrigues, and Carolina Arzelán are full-time, salaried employees of AbbVie and may own AbbVie stock or stock options.

Paula C. Luna received honoraria or fees for serving on advisory boards, as a speaker, or as a consultant, and grants as an investigator, from AbbVie, Amgen, BI, Lilly, Janssen, Pfizer, Novartis, Raffo, and Sanofi. She serves as a doctor at Hospital Aleman, which receives research funds from AbbVie, BI, and Pfizer.
